# Kate Mountstephen

**Published:** 1887-09-24

**Authors:** C. G. Furley


### Kate Mountstephen.

By C. G. Furley.

(Concluded from p. 420, vol. ii.)

### Chapter V.—The Hospital on Fire.

Before midnight, on the evening of little Frank's arrival in the children's ward, both nurse and patient were asleep, worn out, the one with pain the other with fatigue. By midnight, too, the night nurse in the children's ward, who ought to have been awake, had fallen asleep in her chair, and arousing herself suddenly from her doze, knocked over a lamp that stood on a table by her side, an accident which resulted in the destruction of nearly the whole of one wing of the building.

A fire is always a serious accident, but a fire in a hospital, where the greater number of the inmates are ill and helpless, must needs be a terrible one; and while nurses were engaged in conveying their patients to a place of safety, and fire-engines were doing their best to prevent the fire spreading beyond the wing where it had originally broken out, no one thought of Sister Katherine and the child she had taken to her own room.

Kate was awakened by the stifling smoke which penetrated the room, and opening the door, saw flames in the corridor, between her and the staircase at the other end. She closed the door hurriedly, and taking up the child in her arms she wrapped him in a blanket, and emerged once more into the corridor. She thought that she could pass through the flames and reach the stair, or at least some window on the other side of the hospital, that which faced the street, where she could hear the voices of firemen and the cries of a quickly gathering crowd; and guessed that the fire-escapes were in use to take those in danger out of the burning building. But the fire was too strong, and beat her back.

"It does not matter much," she said to herself; "they will remember soon that I am here, and bring an escape round, though I don't know how they could get it into this courtyard. But it is only the second story; a ladder would be long enough."

She waited for a few minutes, which seemed of endless length, listening and looking eagerly for some sign of help, but none came, and the smoke was thickening every instant.

"I must be prepared for the worst, and help myself as best I can," she said.

She knotted the sheets and quilt together to make a rope, and fastened one end to the iron bedstead. Then she tied the child on her back, somewhat in the fashion of an Indian papoose. He was crying with fear and pain, and it was difficult to make him believe there was a reason for her actions without frightening him more. He shrieked aloud as she began to descend the rope, and clutched Kate round the neck so tightly as almost to choke her. Happily it was on that floor the fire had broken out, so there was no danger of their being scorched in their descent; but, as Kate soon found, the rope was too short, and she would be obliged to drop eight or ten feet to reach the ground.

"Frank," she said, as for a minute she hung swinging at the end of the rope, "I must let myself fall to the ground. It will probably hurt your leg. Will you try to be a brave boy, and bear it without crying much?"

"I try," answered Frank, doing his best to suppress the sobs of terror that were shaking his whole body.

Then Kate let go her hold on the rope, trying to throw herself forward, so that whatever befell her, the boy might sustain no further injury. There was one moment, during which she imagined she had sustained a bad "cropper" when following the hounds, and then all was dark.

She did not know that a week had passed before she awoke to life again, in an unfamiliar luxurious room. Past and present were still mingled in her mind; but her surroundings gave her no clue, true or false, to what had happened; this room, with its costly furniture and appointments, was neither her simple hospital chamber nor the dainty apartment which had been Kate Mountstephen's at Coteswold. She recognised the familiar face of Nurse Elliot, but it brought to her mind no memory of the immediate past.

"Where did I come off, nurse?" she asked. "Was it at Three Beech Corner? That was always a nasty jump. Somehow I don't remember how I fell this time."

"My dear, what are you talking of?" exclaimed the nurse. "Don't you remember the fire at the hospital, and your throwing yourself over the window?"

For a moment Kate looked bewildered, but soon her memory became clear. "Yes, yes, I remember," she said. "And the child, little Frank, is he safe?"

"Yes, quite safe, thanks to you. And who do you think he is? The son of that Mr. Barnett, whose wife I attended a year ago. You told me at the time that you knew him."

"His child! Harry Barnett's child!"

"Yes. I recognised him at once when we found the two of you, and we communicated with the father. You may imagine how grateful he is to you. He insisted on your being brought to his house, which we were very glad to do, for, as you may guess, there are no beds to spare in the hospital at present."

Kate raised herself in bed for a moment, though the next she had to fall back on her pillow with a sigh of weakness.

"And is it in his house that I am now?" she demanded.

"Yes."

"You must get me removed at once, to the hospital, to a lodging—anywhere."

"Impossible, Sister. You're not fit to be moved; neither the doctor nor Mr. Barnett would permit it. Mr. Barnett is most anxious for the time to come when you will be able to see him, and receive his thanks."

A faint brief flush came into Kate's pale cheeks. "Does he know who I am?"

"Of course he does."

"But I mean, by what name does he know me?"

"Why! as Sister Katherine."

"Ah!" said the patient, sadly, turning her face to the wall; "it is not Kate Mountstephen he wants to see."

It became her one desire during her convalescence to get away from Harry Barnett's house without seeing him. She felt that an interview would be painful for her, and might, considering their last parting, be embarrassing for him. But when you owe a man hospitality, and he owes you a greater obligation still, it is impossible to refuse to meet him without giving a valid explanation, which explanation it would have been more painful still for Kate to offer to those around her. So, the day before she was to go to Eastbourne to complete her recovery, she braced her courage to meet her former lover.

"After all, why should I hesitate about it?" she said to herself as she waited for him in the fanciful, luxurious boudoir which had been furnished in accordance with Evelyn Ellis's extravagant taste. "Six years is a long enough time to permit of his forgetting all the past, and of my seeming to forget it; and I can trust to my calmness not deserting me when I see him. He may not remember me at all,—I have changed in these years, grown thin and pale and old, and now there is this scar on my brow to help my disguise, and this nun-like hospital gown to make me uglier."

Yet Sister Katherine used to say she liked the hospital uniform. And when she dressed herself on the day Mr. Barnett was to see her she looked at the scar on her face—the result of her fall on the night of the fire—very long and sadly; and sighed as she said, "Ah! he will not know me!" when she turned away from the glass.

"Sister Katherine?" said Harry, interrogatively, as he came into the room, and saw the tall, slender figure; but when he came nearer to hold out his hand and thank her for what she had done, his tone changed,—"Kate, my Kate, can it be you?" he exclaimed.

"It is Kate Mountstephen at least, Mr. Barnett," answered Sister Katherine coldly; but Harry did not heed, did not notice her coldness, being full of his own surprise and delight.

"To think of my finding you here!" he went on. "To know that it is you who saved my boy's life! And I had begun to lose hope of ever seeing you again."

"I don't think you tried very hard to find me," she said, still frosty in manner.

"I did what I could. I wrote to your father's successor at Coteswold, and asked him if he knew your present address. He told me that you had gone to London some years before, but that he did not know where you now lived."

"How could he say such a thing! He was aware of my coming to this hospital, and might at least have referred you to the matron here. But he did not like me. Still, if you had only gone to old Willis—she is settled in Coteswold,—or to my aunt."

"I did not know where either of them was. And Mitchell, the only other person I could have asked about you, was killed, as I suppose you know, in Egypt three years ago."

"But you did try to find me," said Kate, unable to get beyond that one satisfying fact.

"Of course I did. All the last year I have been trying to discover some trace of you. And I meet you here, where I least expected to see you."

"Yes, the unexpected always happens." Deep emotion does not always find fitting words, and in our moments of intensest feeling we are most apt to drift into commonplace.

"But since the unexpected has happened, dearest, what does it mean for you and me?" Harry went on.

"Need it mean anything?"

"Surely it must,—unless, indeed, some one has taken my place in your heart?"

"No, not that; but you yourself——"

"Dear," he said in tender reproach, "do you think that a man who has once known and loved you can ever be happy without you? For a year I have been looking for you, though I hardly dared to think that you could have kept even a memory of me in your heart. Against all reason I did hope that it might be so; and then I could not find you."

"But now," she said softly, "you have found me."

"Have I indeed?—found you for good, in such a way that I may make you mine and keep you for ever?"

An unfamiliar mischievous light flashed for a moment into Sister Katherine's eyes. "Those that find, keep,—don't the children say?" she answered.

Half an hour afterwards, "Harry," she asked, "is it true that you showed Frank a portrait of me? He told me so when they brought him to the hospital; but I did not believe it, for I did not know him to be your son; and besides, I never gave you one."

"No, but poor Mitchell had one, and—and to tell the truth, I stole it out of his album. Was it a very heinous crime?"

"I don't know about the absolute right or wrong of the matter, but I at least cannot help forgiving it," answered Kate smiling.

"But, dearest, I shall not need to content myself with a mere photograph much longer, shall I? You are mine now."

"Yes, Harry, yours now; yours and little Frank's."

To which Harry added:—"To be for ever, what you have been from the beginning, guardian and saviour of both father and son."

